# Antimicrobial Activity and 70S Ribosome Binding of Apidaecin-Derived Api805 with Increased Bacterial Uptake Rate

**DOI:** 10.3390/antibiotics11040430

**Published:** 2022-03-23

**Authors:** Tobias Ludwig, Andor Krizsan, Gubran Khalil Mohammed, Ralf Hoffmann

**Affiliations:** 1 Institute of Bioanalytical Chemistry, Faculty of Chemistry and Mineralogy, Universität Leipzig, Deutscher Platz 5, 04103 Leipzig, Germany; tobias.ludwig@uni-leipzig.de (T.L.); andor.krizsan@uni-leipzig.de (A.K.); gubran.mohammed@uni-leipzig.de (G.K.M.); 2 Center for Biotechnology and Biomedicine (BBZ), Universität Leipzig, Deutscher Platz 5, 04103 Leipzig, Germany

**Keywords:** proline-rich antimicrobial peptide, apidaecin, bacterial ribosome, novel mode of action

## Abstract

In view of the global spread of multiresistant bacteria and the occurrence of panresistant bacteria, there is an urgent need for antimicrobials with novel modes of action. A promising class is antimicrobial peptides (AMPs), including them proline-rich AMPs (PrAMPs), which target the 70S ribosome to inhibit protein translation. Here, we present a new designer peptide, Api805, combining the N- and C-terminal sequences of PrAMPs Api137 and drosocin, respectively. Api805 was similarly active against two *Escherichia coli* B strains but was inactive against *E. coli* K12 strain BW25113. These different activities could not be explained by the dissociation constants measured for 70S ribosome preparations from *E. coli* K12 and B strains. Mutations in the SbmA transporter that PrAMPs use to pass the inner membrane or proteolytic degradation of Api805 by lysate proteases could not explain this either. Interestingly, Api805 seems not to bind to the known binding sites of PrAMPs at the 70S ribosome and inhibited in vitro protein translation, independent of release factors, most likely using a “multimodal effect”. Interestingly, Api805 entered the *E. coli* B strain Rosetta faster and at larger quantities than the *E. coli* K-12 strain BW25113, which may be related to the different LPS core structure. In conclusion, slight structural changes in PrAMPs significantly altered their binding sites and mechanisms of action, allowing for the design of different antibiotic classes.

## 1. Introduction

The global threat of multi- or panresistant bacteria overcoming antibiotic treatments has been discussed for many years, culminating with a 2014 report stating that the death toll of infections with resistant bacteria could rise to 10 million people every year by 2050 [1]. This pessimistic scenario was controversially discussed; as of 2019 the global burden associated with drug-resistant infections was estimated at 4.95 million deaths, of which 1.27 million were directly related to drug resistance [2]. This indicates the risk that humans might face in a post antibiotic era triggered by bacteria virtually resistant against all clinically approved antibiotics. Besides small molecules, antimicrobial peptides (AMPs) are an interesting class of molecules that should be further investigated for their pharmacological promise [3,4]. Although many AMPs attack bacterial membranes (lytic mechanisms), some AMP classes specifically target intracellular bacterial structures, such as proline-rich AMPs (PrAMPs), targeting the 70S ribosome using distinct mechanisms and inhibiting protein translation [5]. PrAMPs are produced in many organisms but not in humans and are typically 18 to 25 residues long in insects and 40 to 60 residues long in mammals [4,6]. They are not cytotoxic to mammalian cells, as they typically enter only bacteria, even when shuttled into cells [7]. PrAMPs are presumed to passively diffuse through the outer membrane of Gram-negative bacteria, including *Escherichia* *coli*, *Salmonella typhimurium*, *Acinetobacter baumannii*, and *Klebsiella pneumonia* [6,8], before they are actively taken up by inner membrane transporters SbmA and MdtM [9,10]. Hence, PrAMPs are inactive or less active against bacterial strains lacking a homolog of SbmA, such as Gram-positive bacteria and strains of *Pseudomonas aeruginosa* [9,11].

The intracellular modes of action of PrAMPs can be classified as apidaecin-like and oncocin-like, based on the mechanisms revealed for short, insect-derived PrAMPs Api137 and Onc112 [12,13]. Api137 was optimized using apidaecin 1b as lead structure, which was identified in *Apis mellifera*, and Onc112 from the Oncopeltus antimicrobial peptide 4 identified in *Oncopeltus fasciatus* [8,14]. Further structural studies using cryo-electron microscopy (cryoEM) showed that Api137 enters the exit tunnel of the 70S ribosome after release of the nascent protein chain and binds to both the 50S ribosomal subunit and the release factor, which is required to release the protein [15]. Thus, release factors RF1 and RF2 are trapped, leading to their rapid depletion due to their low copy numbers in bacterial cells, i.e., ribosome-to-RF1 and -RF2 ratios of 200 and 25, respectively [16]. The lack of free RFs leads to stalling of ribosomes at the stop codon without release of the nascent polypeptide chain, increasing the likelihood of a stop-codon readthrough [15]. Onc112 acts on translation initiation by preventing aminoacyl-tRNA from binding to the peptidyl transferase center of the ribosome [11,17].

Initially, we aimed to design analogs of Api137 (Table 1) that can be produced in high quantities using ribosomal expression in *E. coli* (manuscript in preparation). Thus, the new designer peptides Api801 and Api805 (Table 1) contained only canonical amino acids. In Api801, we substituted the guanidated ornithine (gu-O) in position 1 with Gly and Ile-6 with Val. In Api805, the C-terminal Leu residue was replaced by four residues corresponding to the C-terminal sequence of drosocin (Table 1). Drosocin, a PrAMP derived from *Drosophila melanogaster*, belongs to the group of apidaecin-like PrAMPs, as binding studies suggest [12]. Interestingly and unexpectedly, Api805 showed different activities against *E. coli* K-12 and B strains, indicating different uptake or target binding and potentially a new mode of action for PrAMPs, which prompted us to initiate this study.

## 2. Results

### 2.1. BW25113 Is Not Susceptible to Api805

All four apidaecin analogs, drosocin, and Onc112 were tested for their activity against *E. coli* strains Rosetta DE3 pLysS and BW25113 (Table 2). Although both strains were equally susceptible to Api137 and Onc112, with MIC values of 2 and 4 µg/mL, respectively, Api88 was twofold and Api801 was fourfold less active against BW25113 than against Rosetta DE3 pLysS. Api805 and drosocin were as active as the other apidaecin peptides against Rosetta DE3 pLysS but basically inactive against BW25113 (MIC = 128 µg/mL).

### 2.2. Api805 Binding to Riboome Extracts of E. coli BW25113 and Rosetta

**Figure 1:**
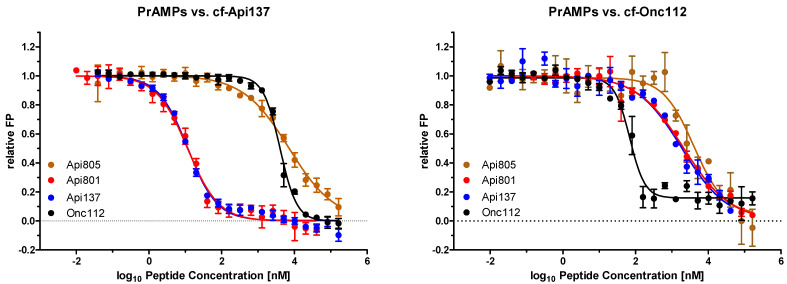
Fluorescence polarization assay testing of Api805 (orange), Api801 (red), Api137 (blue), and Onc112 (black) in competition with cf-Api137 (left) and cf-Onc112 (right) for the ribosomal binding sites of Api137 and Onc112. Experiments were performed twice in triplicate. Error bars indicate the standard deviation of all six replicates. The horizontal dotted line separates positive and negative ranges on the *y*-axis.

The different activity of Api805 in *E. coli* BW25113 and Rosetta was elucidated by studying the binding to the corresponding ribosome extracts (Table 1, Supplementary Materials Figure S1A,B). The K_d_ values of Api805 were 0.557 µmol/L for *E. coli* BW25113 and 0.515 µmol/L for *E. coli* Rosetta compared to 0.382 µmol/L and 0.198 µmol/L, for Api137 and 0.027 µmol/L and 0.051 µmol/L, respectively, for Onc112. As K_d_ values of all tested apidaecin and oncocin analogs were very similar, ribosome binding does not explain the different susceptibility of both *E. coli* strains to Api805. Thus, the binding sites of Api805 and Api801 were probed in an inhibitory assay against the known binding sites of Api137 and Onc112 using the corresponding cf-labeled peptides and the *E. coli* BW25113 ribosome extract (Figure 1, Table 2). The K_i_ values of Api801 and Api137 competing with cf-Api137 were very similar, indicating that they occupy the same binding site (K_i_ = 0.002 µmol/L). In contrast, Api805 was a much weaker competitor (K_i_ = 1.070 µmol/L). Similarly, both Api137 and Api801 inhibited the binding of cf-Onc112 better than Api805 (K_i_ = 0.489 µmol/L). This suggests a different binding site for Api805 at the 70S ribosome compared to the known binding sites of Api137 and Onc112.

In an attempt to define and localize the binding site of Api805 on the 70S ribosome, we tested Api137, erythromycin, and chloramphenicol binding to the exit tunnel of the 50S subunit and kanamycin, known to bind to 30S subunit. However, none inhibited the binding of cf-labeled Api805 (Figure S2). Even more astonishing was that Api805 did not inhibit the binding of cf-labeled Api805 and that Api88 did not inhibit the binding of cf-labeled Api88 (Figures S2 and S3). Thus, it was impossible to map the binding sites of Api805 and Api88 on the 70S ribosome, although the K_d_ values of cf-Api805 and cf-Api88 were in the nanomolar range (Table 2).

### 2.3. Api805 Inhibits Protein Translation

**Figure 2:**
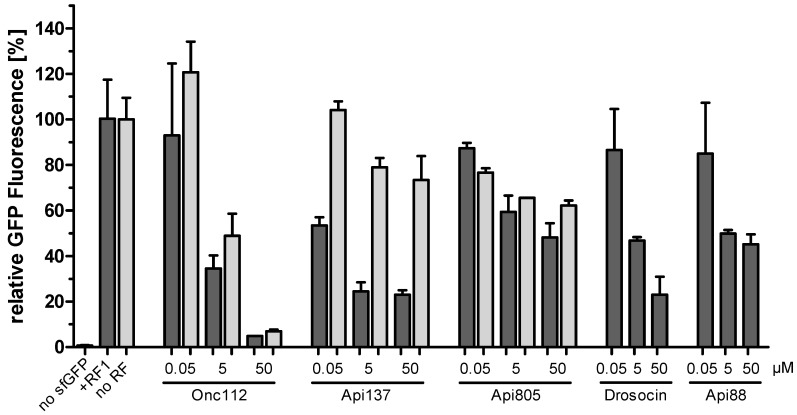
In vitro translation of GFP in the presence of PrAMPs Onc112, Api137, Api805, drosocin, and Api88 with (dark grey) or without (light grey) added release factor RF1. Presented is the measured GFP fluorescence relative to the control experiment without the addition of a PrAMP (=100%). Experiments were performed twice in duplicate. Error bars indicate the standard deviation of all four replicates. Significant differences were determined using *t*-test (Tables S4 and S5).

Next, we investigated whether Api805 inhibits protein translation in vitro by recording the fluorescence of expressed GFP (Figure 2). As Api137 inhibits protein translation by trapping release factors RF1 and RF2, the fluorescence was measured in the absence of release factor and in the presence of RF1 or RF2. We used a ribosome concentration of 2.4 µmol/L, which is ~100-fold higher than the ribosome concentration in *E. coli* [21]. The fluorescence steadily increased, reaching a plateau after around two hours in all three experiments (Figure S4). The highest fluorescence and thus most likely the highest GFP production was observed for RF1, whereas RF2 and no release factor provided 34.5% and 69.5% lower GFP expression levels, respectively. Unexpectedly, the high expression levels for RF1 indicate that protein expression was not stalled, although RF1 should dissociate only slowly from the post-release ribosome without RF3 [22]. Due to the higher expression rate, RF1 was added in the following experiments. Furthermore, RF2 of PureExpress^®^ was isolated from an *E. coli* K-12 strain that carries an A246T substitution, reducing its susceptibility to apidaecin [15,23]. The inhibition was measured for peptide concentrations of 0.05, 5, and 50 µmol/L relative to the fluorescence obtained without a PrAMP (100%).

Protein translation was strongly inhibited by Onc112, independent of the presence of RF1, in a dose dependent manner by up to ~95%, as expected based on its mechanism of action (Figure 2) [17]. In the presence of RF1, protein translation was inhibited by Api137 by ~77% at the highest concentrations and showed only a slight inhibition in the absence of RF1, leading to a similar basal level of GFP production, considering the different initial fluorescence levels (Figure S4). This confirms the proposed mechanism, i.e., Api137 captures RF1 at the ribosome, depleting the bacterial cells for RF1 and consequently inhibiting protein expression [24]. Drosocin showed an effect similar to that of Api137 in the presence of RF1 and was thus not investigated further in the absence of RF1. 

Interestingly, Api805 inhibited GFP expression at similar dose-dependent degrees in the absence and presence of RF1, reducing protein expression by ~38% and ~52%, respectively, at the highest concentration. This indicates a mechanism of action targeting the 70S ribosome but independent of RF1 and, most likely, RF2. A similar inhibitory effect was observed for Api88 in the presence of RF1.

### 2.4. Bacterial Uptake of Api805

**Figure 3:**
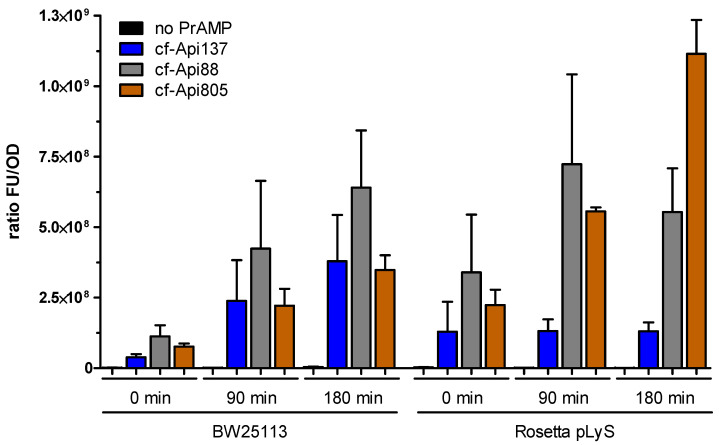
Uptake assay of *E. coli* BW25113 and Rosetta cultures incubated in the absence of PrAMP (black, negative control) or in the presence of cf-labeled Api137 (blue), Api88 (gray), and Api805 (orange) for 0, 90, and 180 min. Presented is the fluorescence remaining in the *E. coli* cells after washing them twice with PBS normalized to the OD_600_ of the corresponding cell culture. The peptide concentration was 23 µmol/L. Experiments were performed twice in triplicate. Error bars indicate the standard deviation of all six replicates. Significant differences were determined using *t*-test (Table S6).

We further investigated the peptide uptake of cf-labeled Api137, Api805, and Api88 in *E. coli* Rosetta and BW25113 to explain the different activity of Api805 (Figure 3). The fluorescence remaining in the bacteria after washing them several times with buffer normalized to the bacterial number (OD_600_ value) continuously increased in *E. coli* BW25113, reaching similar values for Api137 and Api805 and twofold higher values for Api88 after 3 h. Interestingly, the normalized uptake of *E. coli* Rosetta differed considerably among the peptides with the lowest uptake observed for Api137, which remained at a similar level at all time points. The uptake of Api88 was similar in both *E. coli* strains, with a faster initial uptake in *E. coli* Rosetta. Api805 entered *E. coli* Rosetta much faster than *E. coli* BW25113, with approximately threefold higher normalized fluorescence after 180 min, which was also twofold higher than for Api88 and fivefold higher than for Api137. Surprisingly, the substantially different uptake rates of Api137 between the two *E. coli* strains did not relate to the comparable MIC values. The higher uptake rate of Api805 by *E. coli* Rosetta and Api88 in both *E. coli* strains could explain their activities against the tested *E. coli* strains, including the differences observed for Api805. This might indicate that the activities of Api805 and Api88 require high intracellular concentrations.

### 2.5. Api805 Uptake in E. coli Cell Lines with a Disturbed SbmA Transporter

The different uptake ratios obtained for Api805 pointed to a possible role of the SbmA transporter used by PrAMPs, including apidaecin, to enter the bacterial cytoplasm [8]. As an SbmA knockout of *E. coli*, Rosetta was not available and the transposon mutagenesis established in our laboratory, which relies on insertion of a chloramphenicol resistance, could not be applied to chloramphenicol-resistant *E. coli* Rosetta, we used *E. coli* BL21 as another B strain carrying the same SbmA transporter as *E. coli* Rosetta. The activity of Api805 was considerably reduced in the obtained *E. coli* BL21 *sbmA*::Tn10^Cm^ strain, where the *sbmA* gene was disrupted by integration of the phage-derived transposon Tn10 (Figure S5). The MIC of Api805 increased from 2–4 µg/mL in the parent *E. coli* BL21 strain to 128 µg/mL in the *E. coli* BL21 *sbmA*::Tn10^Cm^ strain (Table 3), indicating a strong influence of the SbmA transporter. Upon transformation of a *sbmA*-containing plasmid (psbmA) into *E. coli* BL21 *sbmA*::Tn10^Cm^ and inducing the expression of SbmA, the MIC value of Api805 decreased 16- to 32-fold, reaching almost the activity of the original strain (Table 3). A sequence comparison (NCBI BLAST^®^, accessed June 2020–February 2022) revealed two single-point mutations of the *sbmA* genes in Rosetta/BL21 compared to BW25113, i.e., A31G and G219T. The corresponding mutations, Thr11Ala and Leu73Phe, in SbmA are both located inside the transport tunnel and are likely to affect the peptide uptake. When an *sbmA*_A31G/G219T-harboring plasmid was transformed into *E. coli* BW25113 Δ*sbmA*, the MIC of Api805 was not altered (Table 3). This indicates that the resistance of *E. coli* BW25113 against Api805 relies on other factors. 

When Api805 was incubated in lysates of *E. coli* strains BW25113 and Rosetta, it was only slowly degraded by bacterial proteases, with ~79% and ~58%, respectively, remaining after 24 h. Thus, the half-life time of Api805 was >24 h, which is slightly longer than that of Api137 (Figure S6). The slightly different degradation rates between the two *E. coli* strains do not explain the significantly different activities of Api805.

## 3. Discussion

Apidaecins are promising lead structures for the development of novel antibiotic classes relying on mechanisms not used by clinically approved antibiotics, thus broadening therapeutic options to prevent or at least delay the feared post-antibiotic era. The new apidaecin analog Api805 was designed by substituting the C-terminal Leu residue of Api137 with the C-terminal Pro-Ile-Arg-Val-sequence of drosocin and guOrn1Gly, as well as Val5Ile substitutions. Based on the high activities of Api137 and drosocin, as well as similar binding sites at the bacterial 70S ribosome [12], we expected a new lead structure using apidaecin- and drosocin-binding modes or a mixed mode. The C-terminal sequence extension was anticipated to modify the binding mode slightly, as the C-terminal carboxylic group of Api137, which interacts with the A76 ribose of the deacetylated P-site tRNA [15], was shifted by three residues. Api805 strongly bound to the 70S ribosome preparations of both *E. coli* strains, with similar K_d_ values as those of Api137 and significantly better than those of drosocin (Table 2). However, the inhibitory assays with cf-Api137 and cf-Onc112 indicated that Api805 only weakly competes with Api137 and Onc112. We previously showed similar properties for Api88, which strongly binds to the 70S ribosome (Table 2) but competes only slightly with Api137 and Onc112, although Api88 and Api137 have the same structure, with the exception of a C-terminal amide in Api88 [12,14]. 

The in vitro translation assay confirmed that Onc112 effectively blocks translation, independent of release factors, whereas the inhibitory effect of Api137 depends on release factors, confirming previous reports [15,17]. Drosocin appears to utilize the Api137-binding site at the 70S ribosome, in agreement with the results of previous publication [12], and an RF1-dependent inhibitory effect on protein translation termination similar to that of Api137. In contrast, Api805 and Api88 reduced GFP expression only slightly, by ~40% to 50% (Figure 2), independent of release factor RF1. The obvious lack of a well-defined binding site in combination with a rather mild and unspecific translation inhibition may indicate that translation-related effects are mere a “multimodal effect”, with PrAMPs such as Api805 and Api88 able to bind to different sites at the 70S ribosome. A similar observation was reported for surface-modified GFP, indicating that positively charged proteins show up to 100-fold lower intracellular diffusion coefficients than negatively charged proteins, as they bind to the ribosome [25]. In case of Api805 and Api88, this effect appears to be triggered by the massive uptake of these cationic peptides. Our experiments indicated comparable uptake levels of Api137 and Api805 in *E. coli* BW25113, suggesting that the different MIC values might be a result of the less specific and weaker binding of Api805 to the 70S ribosome. Assuming a volume of 4 fL per *E. coli* BW25113 cell [26], the cellular concentrations of Api137 could reach up to 0.8 mmol/L, which corresponds roughly to 2 g/L [27]. In Rosetta, the uptake of Api805 was threefold higher, indicating intracellular concentrations of ~2.4 mmol/L. This correlates to a 100,000-fold excess of Api805 compared to ribosomes in the cell [21], assuming that all peptides indeed enter and remain in the cytoplasm and do not stick to membranes or other negatively charged cellular components, for example, DNA. Even considering further binding sites, the peptide concentrations would most likely exceed the highest peptide concentration used in the in vitro assay (50 µmol/L), which is equivalent to a peptide-to-ribosome ratio of ~20.

Because the uptake of Api88 is also two- to threefold higher than that of Api137 in both *E. coli* strains, it might also compensate for its weaker ribosome binding, which could explain the equal antimicrobial activities of Api88 and Api137, which are both more active than drosocin and Onc112. We would like to stress that the use of a bulky hydrophobic dye, such as 5(6)-carboxyfluorescein, accounting for roughly 20% of the total molecular weight, might significantly influence bacterial uptake and also favor membrane interactions, leading to overestimated cytosolic peptide concentrations. Thus, it remains unknown whether the proposed “collateral damage” is an important part of the antibacterial mechanism of action.

The identification of SbmA as the main transporter for Api805 once again confirms that the activity of most PrAMPs depends on this transporter. Interestingly, SbmA has recently been implicated as a defense mechanism against membrane-permeabilizing peptides [28] by facilitating their fast uptake into the cytoplasm to accelerate their intracellular degradation in order to prevent lethal damages on the outer membrane. Therefore, hijacking of this transporter-dependent resistance mechanism by PrAMPs targeting the ribosome appears to be a very interesting mode of action. This might also explain the synergistic effects we observed for lytic peptides and PrAMPs in a checkerboard assay [29]. Api137 appeared to depend less on the SbmA transporter, except for *E. coli* BW25113 Δ*sbmA* in 33% TSB medium (Table 3), which confirms a previous report [27,30]. The reason remains unclear, but it suggests that at least some PrAMPs are capable of passing through the inner membrane, utilizing other transporter systems, such as MdtM [10] or passive diffusion.

The reason for this massive influx of Api805 into *E. coli* B strains Rosetta and BL21 and the lower uptake in *E. coli* K-12 strain BW25133 remains unknown. Although *E. coli* B and K-12 strains share over 99% sequence similarity in ~92% of their genomes, their LPS structures are significantly different. The LPS core of *E. coli* K-12 forms its own group, whereas *E. coli* B strains belong to the R1 group, despite having an IS1 insertion interrupting the *waaT* gene. Interruption of *waaT*, which encodes for a galactosyltransferase, leads to a smaller LPS core oligosaccharide of only two hexose units [31,32]. The reduced LPS core might allow PrAMPs to diffuse through the outer membrane of *E. coli* B strains more easily [33]. The increased permeability neither alters the membrane potential nor impairs the vitality of the strain [34]. For example, the uptake of lytic cationic AMPs, such as CAP18, melittin, indolicidin, and cecropins partially depends on the LPS core structure, with knockout mutants of BW25113 being more susceptible [35]. Thus, we hypothesize that the different uptake rates of Api805 in *E. coli* B and K-12 strains are related to the LPS core structure. The reduced LPS core of *E. coli* B strains might facilitate lateral diffusion of PrAMPs, most likely by displacement of divalent cations, such as Mg^2^- and Ca^2+^-stabilizing neighbored LPS molecules [35]. Hence, Api805 may accumulate in the B-strain periplasm and serve as preferred substrate for SbmA, which might explain the SbmA-dependent activity in BL21 compared to BW25113. Thus, it might be useful to consider such a possible resistance mechanism for the design of optimized PrAMPs.

Taken together, the binding site of Api805 remains elusive, and we can only speculate that the binding modes of Api805 and Api88 differ from those of Api137, Onc112, and drosocin, although the binding site appears to partially overlap. This clearly indicates that even slight structural changes alter the mechanism of action of PrAMPs, which is also exemplified by Api88, considering the data reported here and in previous publications [14,18]. We speculate that this variety of alternative mechanisms at the bacterial 70S ribosome explains the impressive evolutionary success story of PrAMPs, as bacteria might not overcome all PrAMPs expressed in different animals at the same time, preventing the spread of bacterial strains resistant against the whole class of PrAMPs. Even slight structural changes, as exemplified by the class of closely related apidaecins, including apidaecins 1a and 1b expressed in honeybees (one Leu18Ile substitution), may allow animals to prevent the spread of multi-PrAMP-resistant bacteria. We further speculate that PrAMPs use a multimodal mechanism, with substitutions favoring one mechanism or another.

## 4. Materials and Methods

### 4.1. Materials and Chemicals

Reagents used are listed in the Supplementary Material File S1. Phage lysate λNK1324 containing transposon Tn10 Cm^R^ carrying a chloramphenicol resistance was obtained from Prof. Dr. Garys Sawers (Universität Halle/Saale). All *Escherichia coli* strains and plasmids used in this study are shown in Tables S1 and S2, respectively. Used primers in are listed in Table S3 and peptides are listed in Table 1. Water was purified with a Purelab Ultra water purification system (electrical resistivity > 18 kΩ·m; organic content < 2 ppb; ELGA LabWater, Celle, Germany).

### 4.2. Peptide Synthesis

Peptides were synthesized on solid phase using a multiple synthesizer (SYRO2000, MultiSynTech GmbH, Witten, Germany), Fmoc/tBu chemistry, in situ activation with DIC in the presence of HOBt, and Rink amide or Wang resins to obtain C-terminal peptide amides or acids, respectively (Table 1). The N-terminus of Api137 was guanidated with HBTU in the presence of NMM [14]. 5(6)-Carboxyfluorescein (cf) was coupled with HBTU in the presence of DIPEA to the free N-termini of all peptides [14], except for guanidated Api137, which was labeled with cf at the δ-amino group of Orn-1. Therefore, Orn1 was incorporated as Fmoc-Orn(Mtt)-OH, and the Mtt-group was selectively cleaved with 2% (*v/v*) TFA and 5% (*v/v*) TIS in DCM. Peptides were cleaved with TFA containing 12.5% (*v/v*) scavenger mixture (ethanedithiol, m-cresol, thioanisole, and water; 1:2:2:2 (by vol)) and precipitated with cold diethyl ether. All peptides were purified by an Äkta Purifier 10 using a Jupiter C18 column (ID 21.2 mm) with an aqueous acetonitrile gradient in the presence of 0.1% TFA as an ion pair reagent. Purities were judged by RP-HPLC using a Jupiter C18-column (ID 4.6 mm or 2 mm). Molecular weights were confirmed by matrix-assisted laser desorption/ionization time-of-flight mass spectrometry (MALDI-TOF-MS; 5800 Proteomic Analyzer; AB Sciex, Darmstadt, Germany).

### 4.3. Antibacterial Activity

The minimal inhibitory concentrations (MIC) were determined at least twice in triplicate using a liquid broth microdilution assay in sterile 96-well plates and a total volume of 100 μL per well. Aqueous peptide solutions were serially 2-fold diluted in 25% MHBII or 33% TSB medium in a volume of 50 µL. An overnight culture was diluted in 25% MHBII or 33% TSB medium to 1.5 × 10^7^ CFU/mL, and an aliquot of 50 μL was added to each well. The final cell count in each well was 7.5 × 10^6^ CFU/mL. The plates were incubated at 37 °C for 20 ± 2 h. The turbidity of each well was measured at a wavelength of 600 nm. The MIC was defined as the lowest peptide concentration where the turbidity was identical to medium only.

### 4.4. Extraction of E. coli Ribosomes

*E. coli* BW25113 and *E. coli* Rosetta pLysS were cultivated in LB medium and harvested at an OD_600_ of ~4 by centrifugation (5000× *g*, 30 min, 4 °C). The cell pellets were washed by resuspension in ribosome preparation buffer (20 mmol/L Hepes-KOH, 6 mmol/L MgCl_2_, 30 mmol/L NH_4_Cl, pH 7.6, 4°C) and subsequent centrifugation (5000× *g*, 30 min, 4 °C). Cell pellets were suspended in ribosome preparation buffer (0.5 g/L) containing freshly added 2-mercapotethanol (4 mmol/L). Lysozyme was added to the resuspended cells and incubated on ice for 30 min. The cell suspension was disrupted six times using a FastPrep-24^TM^ 5G instrument (MP Biomedicals, Solon, OH, USA) with BigPrep 50 mL setting (40 s, 4.0 m/s), incubated with DNase I (5 mL/L) on ice for one hour and centrifuged (16,000× *g*, 30 min, 4 °C). The cell debris was removed from the supernatant by centrifugation (32,000× *g*, 60 min, 4 °C), and the supernatant was centrifuged again (165,000× *g*, 17 h, 4 °C). The pellet was resuspended in ribosome preparation buffer (0.1 mL per g cell pellet) to obtain the 70S ribosome extract and stored at −80 °C. The ribosome concentration was estimated by recording the absorbance at 260 nm (1 AU for a ribosome concentration of 28 nmol/L). The molecular weight of the 70S *E. coli* ribosome was assumed to be 2.3 MDa.

### 4.5. Fluorescence Polarization

K_d_ was determined using a ribosome extract and cf-labeled peptides in ribosome preparation buffer. Briefly, black 384-well plates (flat bottom, Greiner Bio-One GmbH) were blocked with casein (0.5%, *w/v*) in phosphate buffer containing 0.05% (*v/v*) Tween 20 at 4 °C overnight and washed three times with phosphate buffer. A 2-fold dilution series of the ribosome extract (20 μL per well) in ribosome preparation buffer was prepared in a black 384-well plate (23 dilution steps). The cf-labeled peptide was dissolved in ribosome preparation buffer (20 μL, 40 nmol/L), added to each well, and incubated at 28 ± 1 °C. After 90 min, the fluorescence polarization was measured on a PARADIGMTM microplate reader (Beckman Coulter, Salzburg, Austria) in the top read position (λexc = 485 nm, λem = 535 nm) in triplicate on at least two different days. Data were fitted to a nonlinear, dose–response logistical transition equation [y= y0 + a/(1 + (x/x0)b)] using the Levenberg–Marquardt algorithm, with dissociation constants (K_d_) represented by the x0 coefficients (GraphPad Prism for Windows v. 5.02, San Diego, CA, USA).

Ki was determined for ribosome extracts using cf-labeled and unlabeled peptides in ribosome preparation buffer. Black 384-well plates were prepared as described above. Unlabeled peptides or antibiotics (20 µL, 656 µmol/L) were added and 2-fold serially diluted in 23 wells in ribosome preparation buffer. A ribosome solution was prepared by adjusting the ribosome concentration to ~90% of the upper K_d_ plateau obtained for the cf-labeled peptide to be studied and added to each well (10 µL). After incubation (28 ± 1 °C, 90 min), the cf-labeled peptide (10 µL, 80 nmol/L) was added and incubated (28 ± 1 °C, 90 min). The fluorescence polarization was measured on a PARADIGMTM microplate reader in the top read position (λexc = 485 nm, λem = 535 nm) in triplicate on at least two different days. Data were fitted to a nonlinear, dose–response logistical transition equation [y= y0 + a/(1 + (x/x0)b)] using the Levenberg–Marquardt algorithm, with the half-maximal inhibition constants (IC50) represented by the x0 coefficients (GraphPad Prism for Windows v. 5.02, San Diego, CA, USA). Inhibition constants, K_i_, were calculated as described by Mathias and Jung [36].

### 4.6. In Vitro Translation Assay

GFP was expressed using an NEB PureExpress Delta RF123 kit (New England Biolabs, Ipswich, MA, USA). The sfGFP DNA template coding for a UAA stop codon was amplified from the pY71sfGFP plasmid by PCR using sfGFP primers (Table S3). Release factor 1 (RF1) included in the kit was 50-fold diluted (*v/v*) in freshly prepared 1x pure system buffer (PSB, 1 mol/L magnesium acetate, 0.5 mol/L K_3_PO_4_ (pH 7.3), 1 mol/L potassium glutamate, 1 mol/L NH_4_Cl, 0.5 mol/L CaCl_2_, 1 mol/L spermidine, 0.1 mol/L putrescine, 0.1 mol/L DTT). GFP was expressed in a freshly prepared mixture of kit solution A (2 µL), kit solution B (1.5 µL), 50-fold diluted RF1 (0.5 µL), sfGFP template (0.1325 pmol, 35 ng) or water as control (0.25 µL), PSB (0.25 µL), and PrAMP (50, 5, 0.05 or 0 µmol/L) dissolved in water (0.5 µL). The reaction mixture was transferred into a black 384-well plate (flat bottom, Greiner Bio-One GmbH), covered with a lid, and incubated in a microplate reader (Gemini EM, Molecular Devices) at 37 °C for two hours. The fluorescence was recorded every 10 min (λexc = 485 nm, λem = 535 nm).

### 4.7. Uptake of cf-Labeled PrAMPs

Bacteria were grown in 25% MHBII medium overnight, diluted fourfold with fresh medium, and incubated to obtain an OD_600_ of ~1. The cultures were adjusted to an OD_600_ of 1, and cf-labeled peptide was added to achieve a final concentration of 23 µmol/L (~64 µg/mL). After 0 min, 90 min, and 180 min, two aliquots (300 µL) of the culture were transferred to two tubes. One tube was immediately stored on ice in the dark. The second sample was centrifuged (10,000× *g*, 4 °C, 3 min), and the supernatant was transferred to a fresh tube and stored on ice in the dark. The pellet was washed with ice cold PBS (300 µL) and centrifuged (10,000× *g*, 4 °C, 3 min), and the supernatant was transferred into a fresh tube. Both the supernatant and the pellet resuspended in 300 µL ice cold PBS were stored on ice in the dark. After 180 min, an aliquot of the unprocessed sample (100 µL) was transferred to a translucent 96-well plate, and the absorbance was measured at 600 nm. Aliquots (100 µL) of the original cell culture, supernatants, wash solutions, and resuspended pellets were transferred into black 96-well plates to measure the fluorescence in a PARADIGM^TM^ microplate reader in the top read position (λexc = 485 nm, λem = 535 nm). Experiments were performed three times on different days. The relative fluorescence units were normalized to the measured OD_600_ values of the cell culture.

### 4.8. Transposon Mutagenesis

*E. coli* strain BL21(DE3) was randomly mutagenized by Tn10 insertion using the lambda phage lysate λNK1324 carrying a chloramphenicol resistance and grown on LB agar plates in the presence of chloramphenicol (15 µg/mL) at 30 °C overnight [37,38]. The resistant colonies were transferred to 25% MHBII agar plates containing Api137 (32 or 64 µg/mL) and incubated again at 37 °C overnight. Genomic DNA was isolated by a Monarch^®^ Genomic DNA purification kit (New England Biolabs, Frankfurt am Main, Germany) from the presumed double-resistant strains, and the sequences flanking the transposon insertion were determined by arbitrary primed PCR using arbitrary primer ARB6 and Tn10 transposon-specific primer IS10-R complementary to the IS10 element of Tn10 transposon [39,40]. The first round of amplification included six cycles of denaturation (95 °C, 30 s), annealing (30 °C, 30 s), and extension (72 °C, 90 s), followed by 30 cycles of denaturation (95 °C, 30 s), annealing (45°C, 30 s), and extension (72 °C, 2 min), as well as a final extension (72 °C, 6 min). The second round of amplification used arbitrary primer ARB2 and primer IS10-R. The PCR consisted of 30 cycles of denaturation (95 °C, 30 s), annealing (52 °C, 30 s), and extension (72 °C, 2 min), followed by a final extension step (72 °C, 6 min). PCR products were purified with the NucleoSpin^®^ Gel and a PCR cleanup kit (Macherey-Nagel, Düren, Germany) and sequenced using primer IS10-R (Eurofins MWG Operon, Ebersberg, Germany), and the corresponding protein was identified in a sequence database (UniProt and NCBI, 2021).

### 4.9. Peptide Stability in E. coli Lysates

Peptides (7.5 µg) were mixed with lysates of *E. coli* Rosetta pLysS or *E. coli* BW25113 (protein concentration ~0.5 g/L) and incubated on an orbital shaker (37 °C, 750 rpm). After 0, 4, 8 and 24 h, aliquots were taken in duplicate or triplicate, and trichloroacetic acid was added to achieve a final concentration of 3% (*v/v*). Samples were incubated on ice (10 min) and centrifuged (13,000× *g*, 5 min). The supernatant was neutralized with aqueous sodium hydroxide (1 mol/L) and stored at −20 °C. Samples were separated on an ACQUITY arc system (Waters GmbH, Eschborn, Germany) using a Jupiter C_18_ column (ID: 2 mm). Elution was achieved using a linear aqueous acetonitrile gradient containing 0.1% (*v/v*) formic acid. The absorbance was recorded on a 2489 UV/VIS detector at 214 nm (Waters GmbH), and mass spectra were acquired online with an ion trap mass spectrometer equipped with an electrospray ionization source (ESI-IT-MS, Esquire HCT, Bruker Daltonics, Billerica, MA, USA). Peptide recoveries and the corresponding metabolites were calculated based on the peak areas (UV) of the initial peptide peak area at 0 min.

## 5. Conclusions

Api805 appears to follow a different mode of action than that of apidaecin-type PrAMPs exemplified by the well-studied Api137. We speculate that Api805 and Api88 use a new type of mechanism, which is not characterized by a well-defined single binding site in the exit tunnel of the 70S ribosome but relies on interactions with different binding sites at the ribosome or ribosome-attached proteins, i.e., a multimodal mechanism based on high intracellular PrAMP concentrations.

## Figures and Tables

**Table 1:** Sequences of all studied PrAMPs. Gu, O, and r denote 1,1,3,3 tetramethyl guanidine, ornithine, and d-arginine, respectively. Residues substituted in Api137 are marked with bold letters.

Peptides	Sequence	Reference
Api137	gu-ONNRPVYIPRPRPPHPRL-OH	[14]
Api88	gu-ONNRPVYIPRPRPPHPRL-**NH_2_**	[18]
Api801	**G**NNRP**I**YIPRPRPPHPRL-OH	This publication
Api805	**G**NNRP**I**YIPRPRPPHPR**PIRV**-OH	
Drosocin	GKPRPYSPRPTSHPRPIRV-OH	[19]
Onc112	VDKPPYLPRPRPPRrIYNr-NH_2_	[20]

**Table 2:** Antibacterial activities, dissociation (K_d_), and inhibition constants (K_i_) of ribosome binding obtained for all PrAMPs included in the current study for *E. coli* strains BW25113 (BW) and Rosetta DE3 pLysS (Ros).

PrAMP	MIC [µg/mL] *		K_d_ [µmol/L] **		K_i_ [µmol/L] ***	
	BW	Ros	BW	Ros	cf-Api137	cf-Onc112
Api137	2	2	0.382 [0.337–0.432]	0.198 [0.179–0.217]	0.002 [0.0015–0.0020]	0.219 [0.105–0.459]
Api801	16	4	n.d.	n.d.	0.002 [0.0015–0.0020]	0.247 [0.172–0.354]
Api805	128	4	0.557 [0.475–0.652]	0.515 [0.442–0.599]	1.070 [0.769–1.489]	0.489 [0.316–0.759]
Drosocin	128	8	n.d.	1.08 ^[a]^	0.009 ^[a]^	1.78 ^[a]^
Api88	2	1	0.906 [0.740–1.108]	1.22 ^[a]^	0.18 ^[a]^	0.11 ^[a]^
Onc112	4	4	0.027 [0.023–0.030]	0.051 [0.044–0.061]	0.606 [0.552–0.666]	0.008 [0.007–0.011]

* MIC values were determined in 25% MHBII medium. ** K_d_ values were determined for cf-labeled peptides using ribosome extracts of BW25113 and Rosetta DE3 pLysS. *** K_i_ values were determined for unlabeled PrAMPs against cf-labeled Api137 and Onc112 using the BW25113 ribosome extract. Confidence intervals (95%) are given in brackets. n.d. = not determined; ^[a]^ values published for *E. coli* BL21 (DE3) RIL ribosome extract [5,12].

**Table 3:** MIC values obtained for Api137 and Api805 against transposon-mutated *E. coli* strains BW25113 and BL21 DE3 using 25% MHBII and 33% TSB medium containing IPTG (0.1 mmol/L).

Strain		MIC (µg/mL)			
		25% MHBII		33% TSB	
		Api137	Api805	Api137	Api805
*E. coli* BL21 (DE3)		4	4	2	2
	*sbmA*::Tn10^Cm^	16	128	16	128
	*sbmA*::Tn10^Cm^ + psbmA	8	16	8	32
	*sbmA*::Tn10^Cm^ + psbmA_A31G/G219T	4	16	8	32
BW25113 Δ*sbmA*		8	>128	64	>128
	+psbmA	4	>128	8	>128
	+psbmA_A31G/G219T	2	>128	8	>128

## Data Availability

The data presented in this study are available on request from the corresponding author.

## Supplementary Materials

The following supporting information can be downloaded at: https://www.mdpi.com/article/10.3390/antibiotics11040430/s1, File S1: Materials and Chemicals. Figure S1: Fluorescence polarization assay using cf-labeled Api137 (blue), Api805 (orange), Api88 (green), and Onc112 (black) as well as ribosome extracts of *E. coli* strains BW25113 (left) and Rosetta (right) to determine the corresponding K_d_ values. Figure S2: Fluorescence polarization assay testing Api805, Api137, chloramphenicol, erythromycin, and kanamycin in competition with cf-Api805 for the ribosomal binding site of Api805. cf-Api137 was tested as control in competition with Api137 or erythromycin. Figure S3: Fluorescence polarization assay testing Api88 and Api137 in competition with cf-Api88 and cf-Api137, respectively, for the corresponding ribosomal binding sites. Figure S4: Fluorescence measured for the expression of sfGFP in an in vitro translation assay in the presence or absence of release factors RF1 and RF2 (no PrAMPs added). No fluorescence was observed in the absence of the sfGFP template. Figure S5: (A) Sequence alignment of *sbmA* from *E. coli* BL21(DE3) and the reverse complement sequence from the arbitrary-primed PCR product of the genomic DNA of *E. coli* BL21(DE3) *sbmA*::Tn10^Cm^. The sequence of the IS10 element of the Tn10^Cm^ transposon next to the IS10-R primer binding site is indicated by red letters followed by the identified *sbmA* sequence. (B) Agarose gel of the PCR products after an amplification of the *sbmA* gene with genomic DNA isolated from *E. coli* strains BL21(DE3) and BL21(DE3) *sbmA*::Tn10^Cm^ using the primers sbmA fwd and sbmA rev. The Tn10^Cm^ transposon was inserted in *sbmA* after 508 bp counting from the start codon, which resulted in a *sbmA* (1–508 bp)–Tn10^Cm^ (1480 bp)–*sbmA* (509–1221 bp) gene in the genomic DNA of *E. coli* BL21(DE3) *sbmA*::Tn10^Cm^. The Tn10^Cm^ insertion in the sbmA gene leads to a truncated and most likely non-functional SbmA protein. Figure S6: Peptide recoveries determined for Api805 (panel A) and Api137 (panel B) in lysates of *E. coli* strains BW25113 (black) and Rosetta (red) for an incubation period of 24 h. Table S1: Bacterial strains and genotype. Table S2: Genetic elements of plasmids used in the current study. Table S3: Sequences of all primers used in this study. Table S4: *T*-test calculations for the sample set of the in vitro translation of GFP with RF1 in the presence of PrAMPs using an unpaired two-tailed *T*-test to judge the significance. Table S5: *T*-test calculations for the sample set of the in vitro translation of GFP without RF in presence of PrAMP using an unpaired two-tailed *T*-test to judge the significance. Table S6: *T*-test calculations for the uptake experiments of cf-labeled PrAMP into *E. coli* BW25113 and Rosetta pLyS using an unpaired two-tailed *T*-test to judge the significance.
