# Supplementary material for: Antimicrobial Activity and 70S Ribosome Binding of Apidaecin-Derived Api805 with Increased Bacterial Uptake Rate
Source: Antibiotics (Basel). 2022 Mar 23;11(4):430. doi: 10.3390/antibiotics11040430 (PMC9025336; doi:10.3390/antibiotics11040430)
Supplement: Supplementary file 1 [file antibiotics-11-00430-s001.zip › antibiotics-1646074-supplementary.pdf]

Supplementary Materials

Antimicrobial Activity and 70S Ribosome Binding of Apidaecin-Derived Api805 With Increased Bacterial Uptake Rate

Tobias Ludwig, Andor Krizsan, Gubran Khalil Mohammed and Ralf Hoffmann

File S1: Material and Chemicals - Reagents S2

Table S1: Bacterial strains S3

Table S2: Plasmids S3

Table S3: Primer sequences S4

Table S4: T-test results – In vitro translation of GFP with RF1 in presence of PrAMP S5

Table S5: T-test results – In vitro translation of GFP without RF in presence of PrAMP S6

Table S6: T-test results – Uptake of cf-PrAMP S7

Figure S1: Fluorescence polarization assay of Api137, Api805, Api88, and Onc112 ..... S8

Figure S2: Competitive fluorescence polarization assay of Api805 and Api137 ..... S9

Figure S3: Competitive fluorescence polarization assay of Api88 and Api137 ..... S10

Figure S4: In vitro translation of GFP S11

Figure S5: Alignment of *sbmA* sequences and agarose gel of the PCR products S12

Figure S6: Degradation of Api805 and Api137 in bacterial lysates S13

File S1: Materials and Chemicals

Reagents were obtained from the following companies unless stated otherwise: AppliChem GmbH (Darmstadt, Germany): Ethidium bromide solution (1%), 4-(2-hydroxyethyl)-1-piperazineethanesulfonic acid (HEPES, >99.5%), tetracycline hydrochloride (≥95%), and tris(hydroxymethyl)aminomethane (Tris); Biosolve BV (Valkenswaard, Netherlands): piperidine (> 99.5%) and dimethylformamide (DMF, peptide synthesis grade); Bio-Rad Laboratories GmbH (Munich, Germany): Precision Plus Protein™ Dual Xtra protein standard; CalbioChemTM (San Diego, USA): Casein (from bovine milk) and guanidinium hydrochloride (>99.9%); Carl Roth GmbH, Karlsruhe, Germany): Agar-Agar Kobe I, ampicillin sodium salt (≥99%), chloramphenicol (≥98,5%), dichloromethane (DCM), dithiothreitol (DTT, ≥99%), lysozyme (≥45 000 FIP U/mg), lysogeny broth (LB) Miller, kanamycin, magnesium chloride (≥99%), nutrient broth, phosphate buffered saline (PBS, pH 7.4), potassium chloride (≥99%), potassium dihydrogen phosphate (≥99%), putrescine (≥ 99 %), sodium dodecyl sulfate (SDS, >99.5%), sodium hydroxide (≥98%) spermidine (≥ 99 %), and trichloroacetic acid (≥99%); Greiner Bio-One GmbH (Frickenhausen, Germany): 96- and 384-well microtiter plates; Honeywell Fluka™ (Seelze, Germany): 1,2-ethandithiole (≥ 98%), thioanisole (≥98 %), Ammonium chloride (≥99.8%), calcium chloride (≥ 99.5 %), and magnesium chloride (≥99%); Iris Biotech (Marktredwitz, Germany): Leucin-Wang resin; MultiSynTech GmbH (Witten, Germany): Rink amide 4-methylbenzhydrylamine (MBHA) resin, 4-benzyloxybenzyl alcohol (Wang) resin, and 2-(1H-benzotriazol-1-yl)-1,1,3,3-tetramethyluronoium hexafluorophosphate (HBTU); Orpegen Pharma GmbH (Heidelberg, Germany) or MultiSynTech GmbH or Iris Biotech: all 9-fluorenylmethoxycarbonyl- (Fmoc) protected amino acids; Phenomenex Inc. (Torrance, CA, USA): Jupiter C18-columns (internal diameter (ID): 21.2 mm, length: 250 mm, particle size: 15 µm, pore size: 30 nm; ID: 10 mm, length: 250 mm, particle size: 5 µm, pore size: 30 nm; ID: 2 mm, length: 150 mm, particle size: 5 µm, pore size: 30 nm); SERVA electrophoresis GmbH (Heidelberg, Germany): Acrylamide/bisacrylamide (30% T, 2.67% C), agarose, ammonium persulfate (99%), Coomassie Brilliant blue G250, glycine (98.5-101%), protease inhibitor mix, TEMED, trypsin (sequencing grade, MS approved), Tween® 20 (pure); Sigma-Aldrich GmbH (Taufkirchen, Germany): Ammonium chloride (≥ 99.5 %), m-cresol (99%), erythromycin, magnesium acetate tetrahydrate (>99%), N,N-diisopropylcarbodiimide (DIC, >98% by GC), N,N-diisopropylethylamine (DIPEA), 5(6)-carboxyfluorescein (cf, for fluorescence), 1-hydroxy-benzotriazole (HOBt, >98%), thioanisole (≥99%), trifluoroacetic acid (TFA, UV-grade for HPLC), TFA (purum) for peptide synthesis, N-methylmorpholine (NMM, >95% GC), triisopropylsilane (TIS), magnesium sulfate (>97%), potassium chloride (>99%), potassium dihydrogen phosphate (>98%), potassium phosphate (≥ 99 %), sodium acetate (>99%), sodium chloride (≥99,5%), disodium hydrogen phosphate × 12 H2O (≥99%), 2-mercaptoethanol (≥ 99%), Müller Hinton broth II (MHBII), Tris-acetate EDTA-Buffer (10x, TAE), and tryptic soy broth (TSB); Thermo Scientific GmbH (Schwerte, Germany): DNase I (RNase-free, 1 U/µL), Phusion High-Fidelity DNA Polymerase (2U/µl), dNTP Mix (2 mmol/L each), and potassium glutamate (≥ 97 %); VWR (Dresden, Germany): Acetonitrile (≥ 99.9%), diethylether (99.9%) and formic acid (≥99%).

Table S1: Bacterial strains and genotype.

Bacterial Strain	Genotype	Reference
E. coli BW25113	F- *Δ(araD-araB)567 ΔlacZ4787*(::rrnB-3) *λ*$^{-}$ *rph-1 Δ(rhaD-rhaB)568 hsdR514*	Keio Collection
E. coli JW0638	BW25113 *ΔsbmA*	Keio Collection
E.coli Rosetta^{TM} pLysS	F$^{–}$ *ompT gal dcm lon*? *hsdS*$_{B}$($r_{B}^{-}m_{B}^{-}$) λ(DE3) [*malB*$^{+}$]$_{K\text{-}12}$(λ^{S}) pLysSRARE(Cm^{R})	Novagen
E. coli BL21 DE3	F$^{–}$ *ompT gal dcm lon hsdS*$_{B}$($r_{B}^{-}m_{B}^{-}$) λ(DE3) [*malB*$^{+}$]$_{K\text{-}12}$(λ^{S})	Novagen
E. coli BL21 DE3 sbmA::Tn10	BL21 *sbmA*::Tn10 (Cm^{R})	This publication

Table S2: Genetic elements of plasmids used in the current study.

Plasmids	Genetic Elements	Reference
psbmA	pNTR-SD, sbmA Amp^{R}	Mobile Plasmid Collection
psbmA A31G	pNTR-SD, sbmA A31G insert, translates to SbmA T11A Amp^{R}	This publication
psbmA G219T	pNTR-SD, sbmA A31G insert, translates to SbmA L73F Amp^{R}	This publication
psbmA A31G/G219T	pNTR-SD, sbmA A31G insert, translates to SbmA T11A/L73F Amp^{R}	This publication
psfGFP	pY71, sfGFP Kan^{R}	Alexander Mankin

Table S3: Sequences of all primers used in this study.

Primer	Sequence	Reference
pNTR fwd	TTAGGATCCTAAGGAGGTGGCC	This publication
pNTR rev	ACTATAGCTGAATTCCCGGCC	
sbmA fwd	GCGAAGATAGAGGATTGACGCG	
sbmA rev	CAGCAGTTGTTAAAACGATAAG	
A31G fwd	TTCCCAAAGCCGGGAGCGTTTTTTCTCTCGG	
A31G rev	CCGAGAGAAAAAACGCTCCCGGCTTTGGGAA	
G219T fwd	AGGACTTTTTGCATTTTTCTGGTTTATCTAC	
G219T rev	GTAGATAAACCAGAAAAATGCAAAAAGTCCT	
sfGFP fwd	TAATACGACTCACTATAGGG	
sfGFP rev	CATGAAGCTTATTTTTCGAACTGCGGAT	
ARB2	GGCCACGCGTCGACTTAGTTAC	[18]
ARB6	GGCCACGCGTCGACTAGTACNNNNNNNNNNACGCC	
IS10-R	CAAGATGTGTATCCACCTTAACTTAATG	

Table S4: T-test calculations for the sample set of the in vitro translation of GFP with RF1 in the presence of PrAMPs using an unpaired two-tailed T-test to judge the significance. One, two, and three asterisks (*) indicate P values of <0.1, <0.01, and <0.001, which were all considered as significantly different.

Sample	P value	P value summary	Significance of difference (P < 0.05)
**+RF1 / Onc112**			
no peptide vs. 0.05 µM	0,5626		No
no peptide vs. 5 µM	< 0.0001	***	Yes
no peptide vs. 50 µM	< 0.0001	***	Yes
0.05 µM vs. 5 µM	0,0043	**	Yes
5 µM vs. 50 µM	< 0.0001	***	Yes
**+RF1 / Api137**			
no peptide vs. 0.05 µM	0,0003	***	Yes
no peptide vs. 5 µM	< 0.0001	***	Yes
no peptide vs. 50 µM	< 0.0001	***	Yes
0.05 µM vs. 5 µM	< 0.0001	***	Yes
5 µM vs. 50 µM	0,3726		No
**+RF1 / Api805**			
no peptide vs. 0.05 µM	0,1697		No
no peptide vs. 5 µM	0,0001	***	Yes
no peptide vs. 50 µM	0,0002	***	Yes
0.05 µM vs. 5 µM	< 0.0001	***	Yes
5 µM vs. 50 µM	0,0199	*	Yes
**+RF1 / Api88**			
no peptide vs. 0.05 µM	0,2400		No
no peptide vs. 5 µM	0,0002	***	Yes
no peptide vs. 50 µM	< 0.0001	***	Yes
0.05 µM vs. 5 µM	0,0353	*	Yes
5 µM vs. 50 µM	0,2126		No
**+RF1 / Drosocin**			
no peptide vs. 0.05 µM	0,1997		No
no peptide vs. 5 µM	0,0001	***	Yes
no peptide vs. 50 µM	< 0.0001	***	Yes
0.05 µM vs. 5 µM	0,0017	**	Yes
5 µM vs. 50 µM	0,0002	***	Yes

Table S5: T-test calculations for the sample set of the in vitro translation of GFP without RF in presence of PrAMP using an unpaired two-tailed T-test to judge the significance. One, two, and three asterisks (*) indicate P<0.1, P <0.01, and P <0.001, which were all considered as significantly different.

Sample	P value	P value summary	Significance of difference (P < 0.05)
**no RF / Onc112**			
no peptide vs. 0.05 µM	0,2645		No
no peptide vs. 5 µM	0,0111	*	Yes
no peptide vs. 50 µM	< 0.0001	***	Yes
0.05 µM vs. 5 µM	0,0003	***	Yes
5 µM vs. 50 µM	0,0003	***	Yes
**no RF / Api137**			
no peptide vs. 0.05 µM	0,8145		No
no peptide vs. 5 µM	0,2473		No
no peptide vs. 50 µM	0,1526		No
0.05 µM vs. 5 µM	0,0002	***	Yes
5 µM vs. 50 µM	0,4227		No
**no RF / Api805**			
no peptide vs. 0.05 µM	0,1992		No
no peptide vs. 5 µM	0,0665		No
no peptide vs. 50 µM	0,0466	*	Yes
0.05 µM vs. 5 µM	< 0.0001	***	Yes
5 µM vs. 50 µM	0,0396	*	Yes

Table S6: T-test calculations for the uptake experiments of cf-labeled PrAMP into *E. coli* BW25113 and Rosetta pLyS using an unpaired two-tailed T-test to judge the significance. One, two, and three asterisks (*) indicate P<0.1, P <0.01, and P <0.001, which were all considered as significantly different.

**Sample**		**P value**	**P value summary**	**Significance of difference (P < 0.05)**
**BW25113**				
Cf-Api137 vs Cf-Api88	0 min	0,0013	**	Yes
Cf-Api137 vs Cf-Api805	0 min	0,0081	**	Yes
Cf-Api88 vs Cf-Api805	0 min	0,0929		No
Cf-Api137 vs Cf-Api88	90 min	0,1349		No
Cf-Api137 vs Cf-Api805	90 min	0,8473		No
Cf-Api88 vs Cf-Api805	90 min	0,1073		No
Cf-Api137 vs Cf-Api88	180 min	0,0343	*	Yes
Cf-Api137 vs Cf-Api805	180 min	0,7231		No
Cf-Api88 vs Cf-Api805	180 min	0,0139	*	Yes
**Rosetta**				
Cf-Api137 vs Cf-Api88	0 min	0,0495	*	Yes
Cf-Api137 vs Cf-Api805	0 min	0,2009		No
Cf-Api88 vs Cf-Api805	0 min	0,2725		No
Cf-Api137 vs Cf-Api88	90 min	0,0011	**	Yes
Cf-Api137 vs Cf-Api805	90 min	< 0.0001	***	Yes
Cf-Api88 vs Cf-Api805	90 min	0,2293		No
Cf-Api137 vs Cf-Api88	180 min	< 0.0001	***	Yes
Cf-Api137 vs Cf-Api805	180 min	< 0.0001	***	Yes
Cf-Api88 vs Cf-Api805	180 min	0,0003	***	Yes
**Cf-Api137**				
BW25113 vs Rosetta	0 min	0,0627		No
BW25113 vs Rosetta	90 min	0,1129		No
BW25113 vs Rosetta	180 min	0,0047	**	Yes
**Cf-Api88**				
BW25113 vs Rosetta	0 min	0,0237	*	Yes
BW25113 vs Rosetta	90 min	0,0960		No
BW25113 vs Rosetta	180 min	0,4265		No
**Cf-Api805**				
BW25113 vs Rosetta	0 min	0,0232	*	Yes
BW25113 vs Rosetta	90 min	0,0003	***	Yes
BW25113 vs Rosetta	180 min	< 0.0001	***	Yes

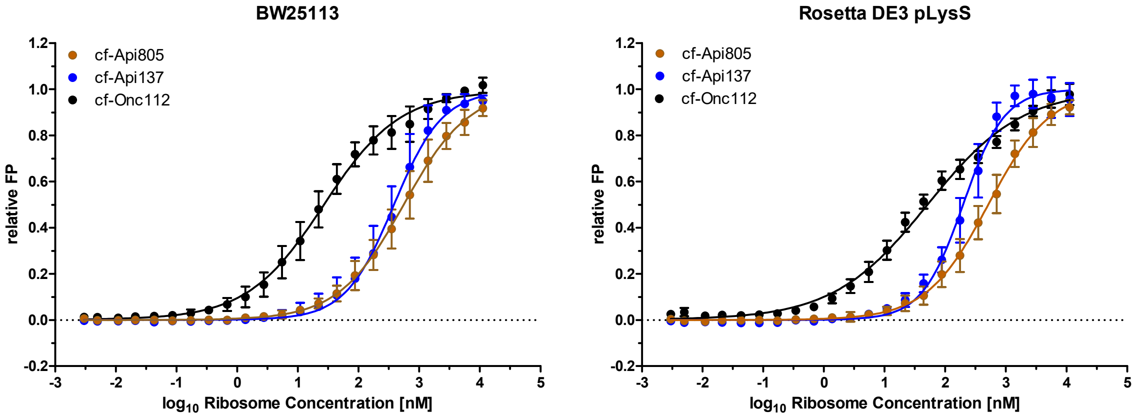

Figure S1. Fluorescence polarization assay using cf-labeled Api137 (blue), Api805 (orange), Api88 (green), and Onc112 (black) as well as ribosome extracts of *E. coli* strains BW25113 (left) and Rosetta (right) to determine the corresponding K_d values. Experiments were done twice in triplicates. Error bars show the standard deviation of all six replicates. The horizontal dotted line separates positive and negative ranges on the Y-axis.

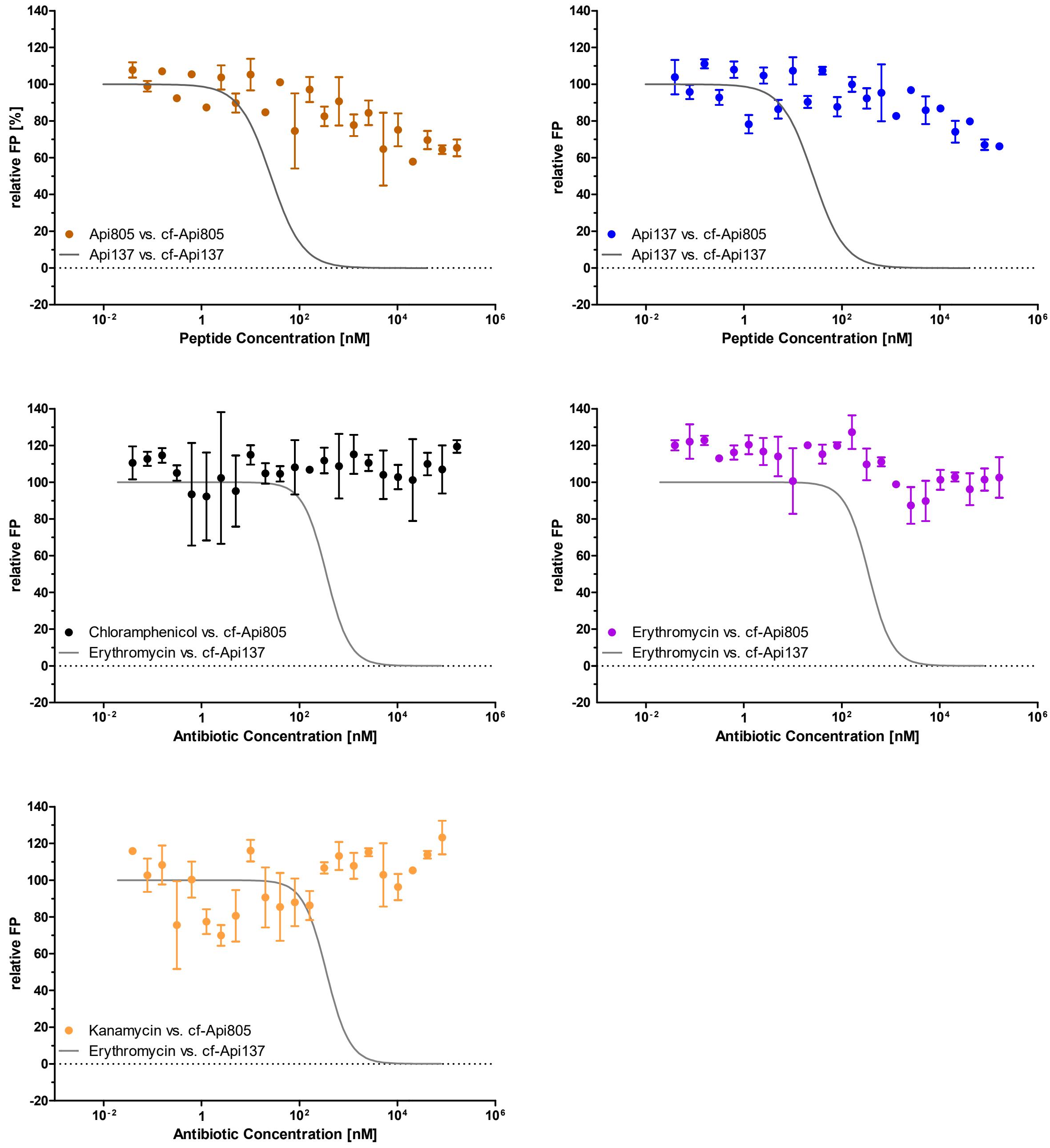

Figure S2. Fluorescence polarization assay testing Api805, Api137, chloramphenicol, erythromycin, and kanamycin in competition with cf-Api805 for the ribosomal binding site of Api805. cf-Api137 was tested as control in competition with Api137 or erythromycin. Experiments were done twice in triplicates. Error bars show the standard deviation of all six replicates. The horizontal dotted line separates positive and negative ranges on the Y-axis.

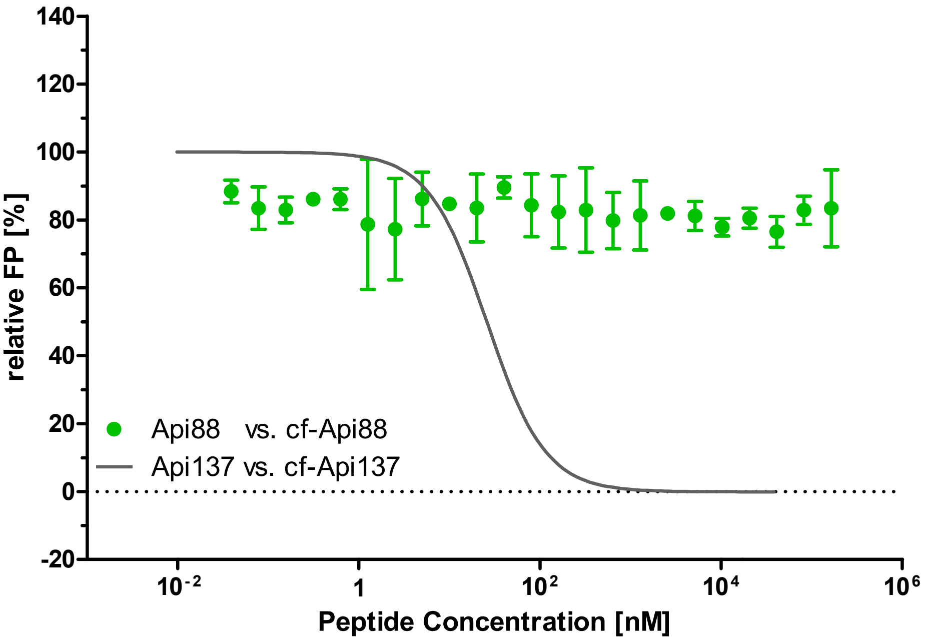

Figure S3. Fluorescence polarization assay testing Api88 and Api137 in competition with cf-Api88 and cf-Api137, respectively, for the corresponding ribosomal binding sites. Experiments were done twice in triplicates. Error bars show the standard deviation of all six replicates. The horizontal dotted line separates positive and negative ranges on the Y-axis.

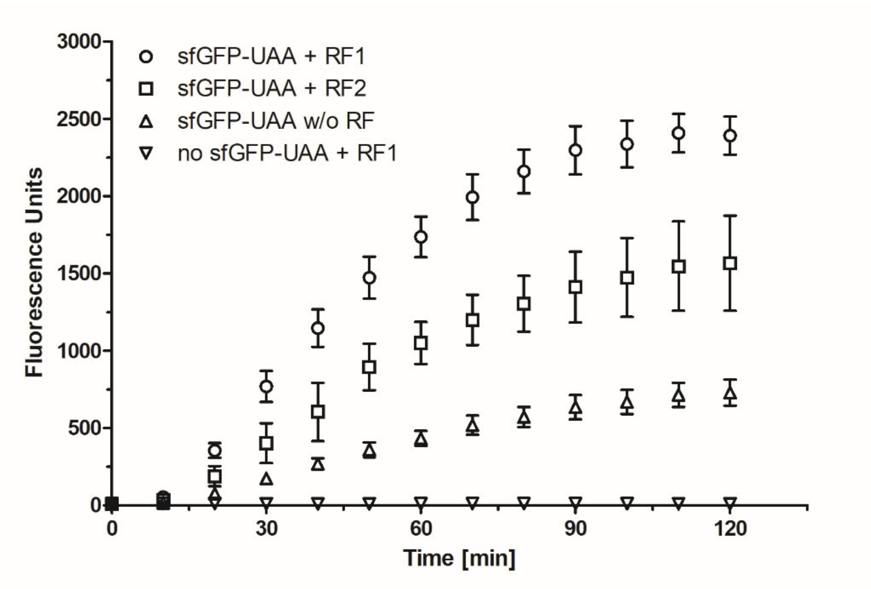

Figure S4. Fluorescence measured for the expression of sfGFP in an *in vitro* translation assay in the presence or absence of release factors RF1 and RF2 (no PrAMPs added). No fluorescence was observed in the absence of the sfGFP template. Experiments were done three times in duplicates. Error bars show the standard deviation of all six replicates.

A

```
sbma::Tn10Cm      --------------------------------------------GGCTGTAGATGGCT--    14
sbmA              TACGCTTACTACATTGTTTGCGTAGGACTTTTTGCATTTTTCTGGTTTATCTACAGCCCG    240
                                                              *  * *  *  **

sbma::Tn10Cm      -------GGCCGC-------AGTATT-AGATGTGGTCGGGGGTCGGGCGGTATTGGGGCA    59
sbmA              CACCGTTGGCAATACTGGTCAATACTCGGTACTGCACTGATCATCTTCGTCACCTGGTTT    300
                         ***          * ** *  *   **  * *        **  *   **

sbma::Tn10Cm      CGCGTCGACTAGTACGGGGTGGCGGACGCCTTGTATGCGCCGTTCTATGATCTGATTCAA    119
sbmA              TTGGTGGAAGTCGGGGTCGCCGTCAACGCCTGGTATGCACCGTTCTATGATCTGATTCAA    360
                     ** **       *  *  *   ****** ****** *********************

sbma::Tn10Cm      ACCGCGCTAAGTTCGCCGCCTAAAGTCACTATCGAACAATTTTATCGCGAACTGTGGGTC    179
sbmA              ACCGCGCTAAGTTCGCCGCATAAAGTCACTATCGAACAATTTTACCGCGAAGTGGGCGTC    420
                  ******************* ************************ ****** ** * ***

sbma::Tn10Cm      TTTCTGGGGATTGCGCTGATCGCTGTGGTGATCAGTGTGCTGAACAACTTCTTTGTCAGT    239
sbmA              TTTCTGGGGATTGCGCTGATCGCTGTGGTGATCAGTGTGCTGAACAACTTCTTTGTCAGT    480
                  ************************************************************

sbma::Tn10Cm      CACTACGTGTTCCGCTGGCGTACGGCGACTGATGAATCCCCTAATGATTTTGGTAA----    258
sbmA              CACTACGTGTTCCGCTGGCGTACGGCGATGAACGAATATTACATGGCGAACTGGCAACAA    540
                  ****************************
```

B

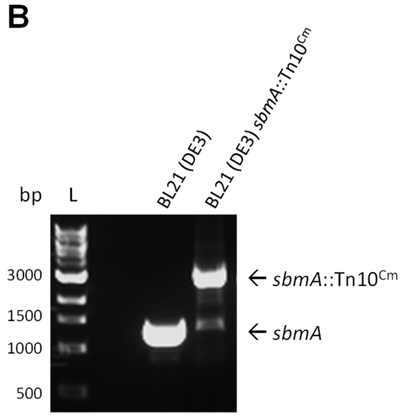

Figure S5. (A) Sequence alignment of *sbmA* from *E. coli* BL21(DE3) and the reverse complement sequence from the arbitrary-primed PCR product of the genomic DNA of *E. coli* BL21(DE3) *sbmA*::Tn10^{Cm}. The sequence of the IS10 element of the Tn10^{Cm} transposon next to the IS10-R primer binding site is indicated by red letters followed by the identified *sbmA* sequence. **(B)** Agarose gel of the PCR products after an amplification of the *sbmA* gene with genomic DNA isolated from *E. coli* strains BL21(DE3) and BL21(DE3) *sbmA*::Tn10^{Cm} using the primers sbmA fwd and sbmA rev. The Tn10^{Cm} transposon was inserted in *sbmA* after 508 bp counting from the start codon, which resulted in a *sbmA* (1-508 bp) – Tn10^{Cm} (1480 bp) – *sbmA* (509-1221 bp) gene in the genomic DNA of *E. coli* BL21(DE3) *sbmA*::Tn10^{Cm}. The Tn10^{Cm} insertion in the sbmA gene leads to a truncated and most likely non-functional SbmA protein.

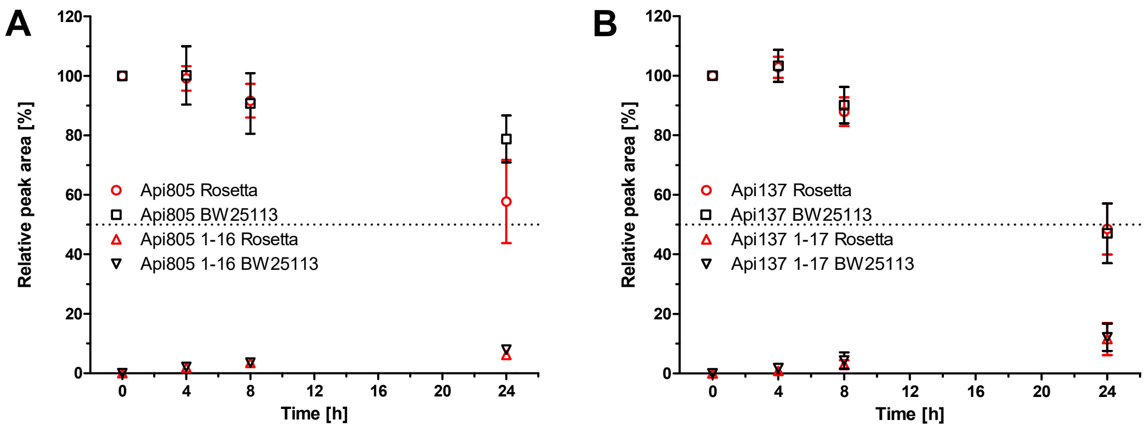

Figure S6. Peptide recoveries determined for Api805 (panel A) and Api137 (panel B) in lysates of *E. coli* strains BW25113 (black) and Rosetta (red) for an incubation period of 24 h. Peptide quantitation relied on the peak areas obtained by RP-HPLC, which were normalized to the peptide quantities obtained for an incubation time of 0 min (set to 100%). Shown are also the major degradation products Api137 1-17 (Gu-ONNRPVYIPRPRPPHPR-OH) and Api805 1-16 (GNNRPIYIPRPRPPHP-OH). Experiments were done twice in duplicates. Error bars show the standard deviation of all four replicates.
